# Decipher the Helicobacter pylori Protein Targeting in the Nucleus of Host Cell and their Implications in Gallbladder Cancer: An *insilico* approach

**DOI:** 10.7150/jca.63517

**Published:** 2021-10-25

**Authors:** Yunjian Wang, Ahamad Imran, Ashwag Shami, Anis Ahmad Chaudhary, Shahanavaj Khan

**Affiliations:** 1 Department of Hepatobiliary and Pancreatic Surgery, The Affiliated Tumor Hospital of Zhengzhou University, Zhengzhou City, Henan Province, 450008, China.; 2 King Abdullah Institute for Nanotechnology, King Saud University, Riyadh 11451, Saudi Arabia.; 3 Department of Biology, College of Sciences, Princess Nourah bint Abdulrahman University, Riyadh 11617, Saudi Arabia.; 4 Department of Biology, College of Science, Imam Mohammad Ibn Saud Islamic University (IMSIU), Riyadh 11623, Saudi Arabia.; 5 Department of Health Sciences, Novel Global Community Educational Foundation, Australia.; 6 Department of Bioscience, Shri Ram Group of College (SRGC), Muzaffarnagar, UP, India.; 7 Department of Pharmaceutics, College of Pharmacy, PO Box 2457, King Saud University, Riyadh 11451, Saudi Arabia.

**Keywords:** *Helicobacter pylori*, Protein targeting, Host cell, Nucleus, Health informatics, Gallbladder cancer

## Abstract

Gallbladder cancer (GBC) is one of the leading causes of cancer-related mortality worldwide. Researchers have investigated that specific strains of bacteria are connected with growth of different types of cancers in human. Some reports show possible implication of *Helicobacter pylori* (*H. pylori*) in the etiology of gallbladder cancer (GBC). Their enigmatic mechanisms, nevertheless, are not still well clear. We sought to predict whether various proteins of H. pylori targeted to nucleus of host cells and their implication in growth of gallbladder cancer. GBC is one of the leading causes of cancer mortality worldwide. We applied bioinformatics approach to analyze the *H. pylori* proteins targeting into the nucleus of host cells using different bioinformatics predictors including nuclear localization signal (NLS) mapper Balanced Subcellular Localization (BaCelLo) and Hum-mPLoc 2.0. Various nuclear targeting proteins may have a potential role in GBC etiology during intracellular infection. We identified 46 *H. pylori* proteins targeted into nucleus of host cell through bioinformatics tools. These *H. pylori* nucleus-targeting proteins might alter the normal function of host cells by disturbing the different pathways including replication, transcription, translation etc. Various nucleus-targeted proteins can affect the normal growth and development of infected cells. We propose that *H. pylori* proteins targeting into the nucleus of host cells regulate GBC growth using different strategies. These integrative bioinformatics research demonstrated several *H. pylori* proteins that may serve as possible targets or biomarkers for early cure and treatment or diagnosis GBC.

## Introduction

Cancer is the second leading cause of death in the United States and a predominant public health problem worldwide 1. Gallbladder cancer (GBC) is the fifth most common gastrointestinal cancer worldwide with poor prognosis 2-4. The number of new cases of GBC and other biliary cancers in the United States was estimated to be approximately 11,740, with 3,830 deaths reported in 2016 1. GBC is most frequently associated with the biliary tract. GBC shows the highest incidence in the sixth and seventh decades of life, and females are affected two to six times more often than males 2, 5, 6. Although GBC is more common in Korea, Japan, Northern India, and Eastern European countries, elevated incidence rates have been observed in Latin America 7. The infections of different types of bacteria are associated with the progress and development of many diseases including typhoid, diarrhea and different types of cancer 8-10. Various factors are involved in the process of carcinogenesis for the growth of different types of cancer such as exposure to specific chemicals, obesity, diet, reproductive factors, hepato-biliary anomalies, cholelithiasis (particularly mixed gallstone or gallstone disease), and poor prognosis, unsatisfactory treatment 4, and late diagnosis of chronic gallbladder infections 11. Similarly different factors have been associated with the growth and progression of GBC. The percentage of patients suffering from GBC after cholecystectomy for assumed gallbladder stone disease is 0.5-1.5% 12. In addition, genetic disorders such as Peutz-Jegher syndrome, anomalous pancreaticobiliary ductal union, and multiple familial polyposis/Gardener syndrome are associated with GBC 13-15. The relationship between life style, genetic predisposition, and previous infection in GBC is not well understood 7. The existence of *H. pylori* and *H. bilis*, both in the bile and gallbladder, was confirmed in more than 75% of patients with GBC and more than 50% of patients with chronic cholecystitis that underwent surgery 16-18. *H. pylori* is a gram-negative, micro-aerophilic, spiral-shaped, flagellated, and slow-growing bacterium and probably the cause of the most common chronic bacterial infections in humans, present in almost half of the world's population 19, 20. KHP30 phage observed to be associated as an episome with NY43 strain of *H. pylori* 21, 22.

Recent reports have indicated the presence of *H. pylori* in the gallbladders and bile of approximately 75% of patients with GBC and about 50% of patients with chronic cholecystitis 18. Although studies have revealed some possible mechanisms involved in biliary carcinogenesis, most key events and specific connections to *H. pylori* infection in this multifaceted cascade that directs the transformation of epithelial cells in the gallbladder remain unknown and require additional investigation. The aim of the present work was to determine *H. pylori* proteins that are localized into the host cell nucleus and their potential associations with GBC. In this study, we focused on the association between chronic *H. pylori* infection and GBC development.

## Materials and methods

### Retrieve the *H. pylori* proteome

We performed various specified searches to retrieve the whole proteome of *H. pylori*. Eventually we were focused to the UniProt (Universal Protein Resource) database to predict the nucleus-targeting proteins of *H. pylori* in the host cell 23. This UniProt database developed through the collection of PIR protein database, SWISS-PROT, and TrEMBL 23-25 contains immense information regarding the *H. pylori* proteome*.* The proteomes of various strains of *H. pylori* such as strains ATCC 700392/26695 and ATCC 27545, are available in these databases 21, 26.

### Selection of a predictive computational tool

The whole proteome of *H. pylori* strain ATCC 700392/26695 was selected for the prediction of nucleus-targeting proteins in human gallbladder cells. We were used different tools including ExPASy Compute pI/Mw tool, cNLS mapper, Balanced Subcellular Localization (BaCelLo) and Hum-mPLoc 2.0 bioinformatics predictor.

### Prediction of pI values and MWs using the ExPASy Compute pI/Mw tool

The ExPASy Compute pI/Mw tool was used to predict the theoretical isoelectric point (pI) and molecular weight (MW) of the query sequence of a particular protein 27. The tool was utilized to access the extensive annotations available in the SWISS-PROT database 24.

### Prediction of NLS in the *H. pylori* proteome using cNLS mapper

We were used cNLS predictor to analyze the possible monopartite and bipartite NLSs in whole protein sequences of *H. pylori* proteome 28. NLS prediction may be used to predict the nucleus-targeting ability of specific proteins 28. The cNLS predictor shows NLS values in the form of an NLS cut-off, and protein sequences with cut-off values of 10 to 8, 7 to 8, 5 to 3, and 1 to 2 were identified as absolutely targeting the nucleus, partly targeting the nucleus, targeting both the cytoplasm and nucleus, and targeting the cytoplasm, respectively. Moreover, protein sequences with cut-off values between two ranges were rounded to the closest whole integer.

### Prediction of protein targeting using the BaCelLo predictor

*H. pylori* proteins targeting the nucleus of the host cell were predicted using BaCelLo 29. This predictor may be used to identify proteins in organisms of three different kingdoms (Fungi, Plants, and Animals). In the current study, we analyzed proteins of the organisms from the animal kingdom. The BaCelLo predictor is a computational tool based on diverse support vector machines (SVMs) structured in a decision tree 29.

### Selection of BaCeILo-predicted proteins using the Hum-mPLoc 2.0 predictor

*H. pylori* proteins targeting the nucleus and other compartments in humans were predicted by utilizing the Hum-mPLoc 2.0 subcellular localization predictor 30. This predictor is based on a top-down approach to increase the power to predict human proteins targeting subcellular components, including the nucleus. Hum-mPLoc 2.0 predicted 14 different classes of subcellular localization, including the nucleus, mitochondrion, cytoplasm, centriole, endoplasmic reticulum, Golgi apparatus, and lysosome, etc.

## Results

### Search for the *H. pylori* proteome

UniProt is a comprehensive database that includes the whole *H. pylori* proteome. We select the ATCC 700392/26695 strain of *H. pylori* because it had the highest number of proteins (1,552) identified among the available proteomes 26.

### Selection of computational tools for the prediction study

**Figure 1:**
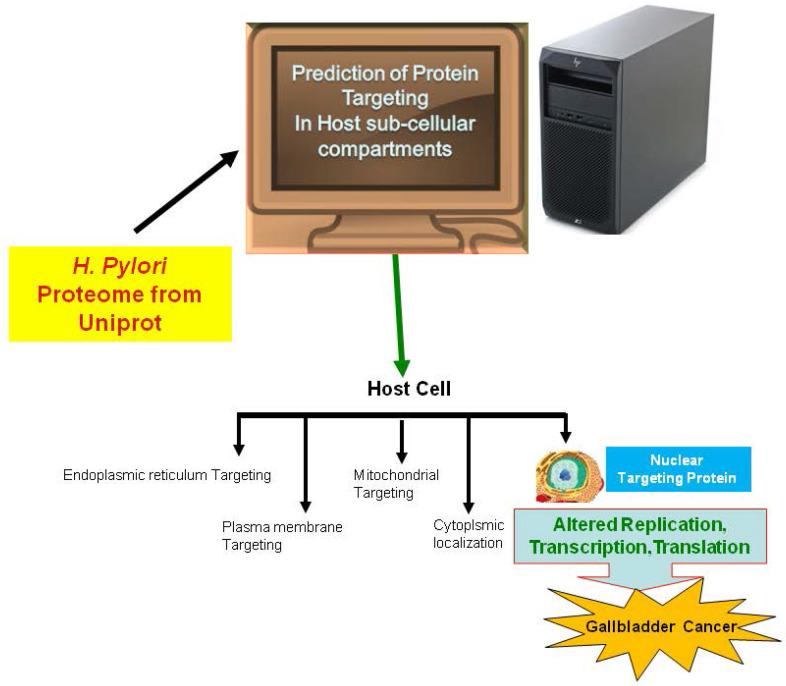
The image shows the method use for the prediction of nuclear targeting proteins using *in silico* approach.

In the current study, we employed the ExPASy Compute pI/Mw tool to predict the pI and MW of proteins, cNLS mapper to determine NLSs, BaCelLo to identify proteins targeting different components of host cells, and Hum-mPLoc 2.0 to predict proteins targeting the nucleus of the host cell because of the relative specificities of their predictive approaches (Fig. 1).

### Prediction of pI values and MWs using the ExPASy Compute pI/Mw tool

**Figure 2:**
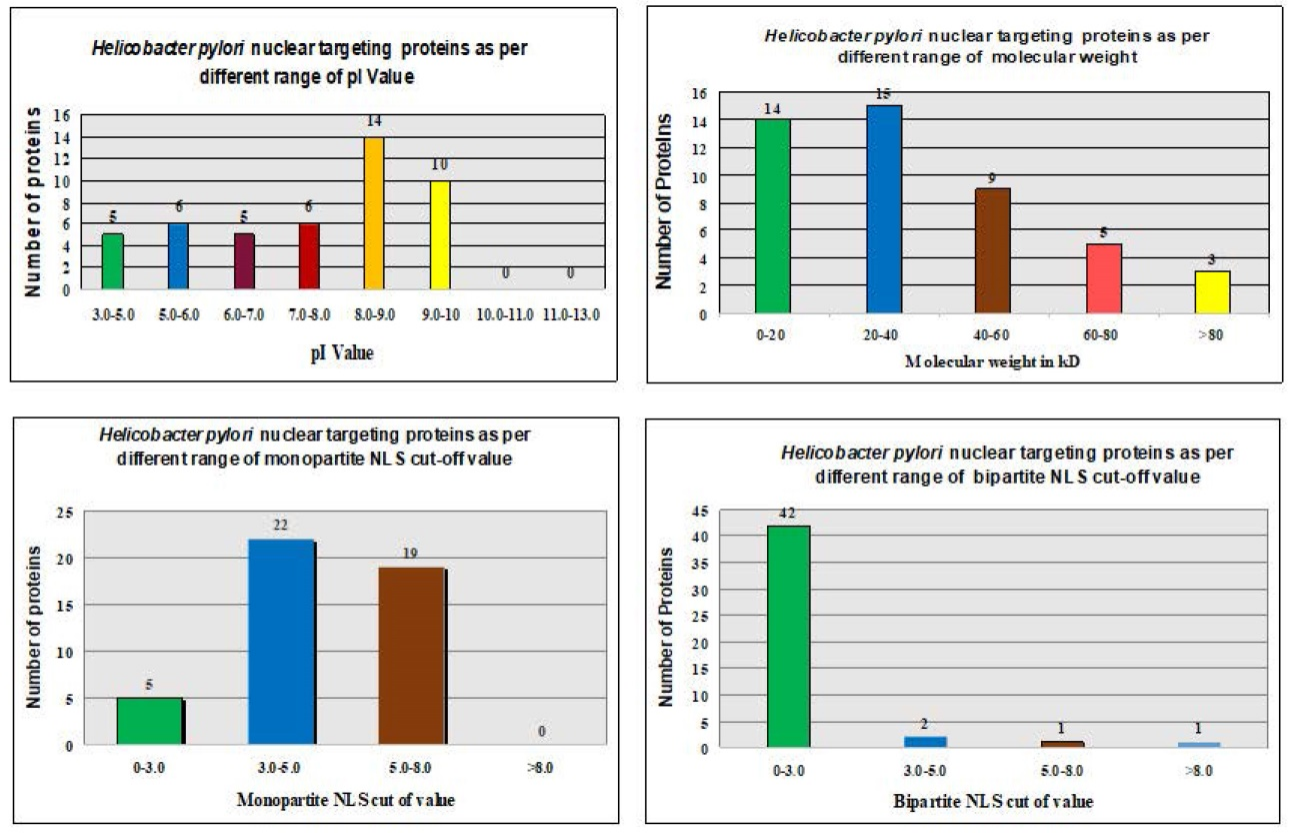
*In silico* analysis of *H. pylori* proteins that target the nucleus of host cells and their relationship to different parameters.

The ExPASy Compute pI/Mw tool calculated theoretical pI values and MWs of proteins in the *H. pylori* proteome (Fig. 2 and Table 1).

The pI values showed no consistent pattern of proteins targeting in the nucleus of the host cell 31, 32. However, the maximum number of nucleus-targeting proteins (14 proteins) was observed in the pI range of 8.0-9.0. Increase in the MWs, consistently decreased the frequency of nuclear targeting proteins, except in the 0-20 kDa range (Fig. 2 and Table 1). The least proteins targeting observed in the nucleus of the host cell with MW > 80 kDa (Fig. 2 and Table 1).

### Prediction of NLSs in the *H. pylori* proteome using the cNLS mapper

**Figure 3:**
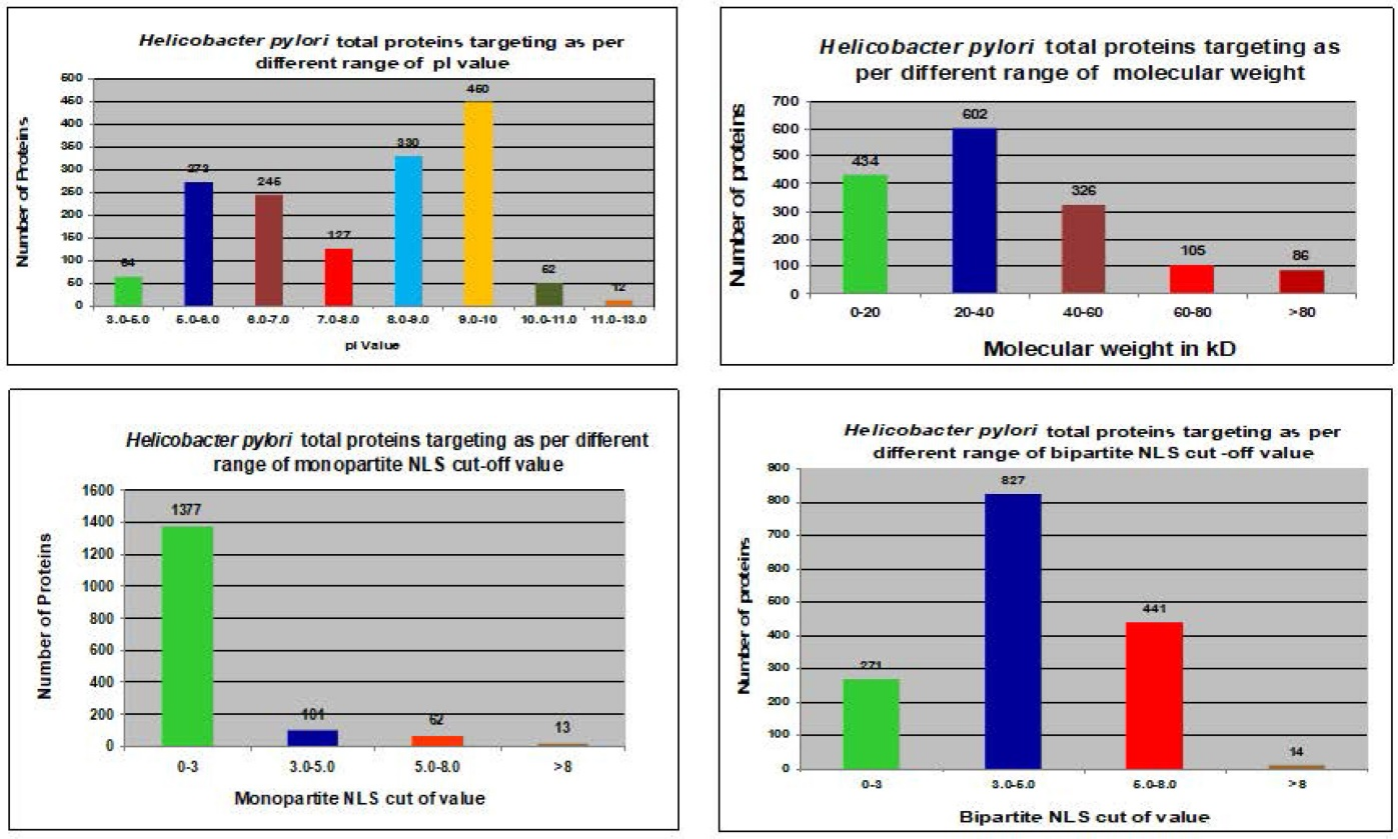
*In silico* analysis of total *H. pylori* proteins in host cells and their relationship to different parameters.

We utilized the cNLS mapper to analyze NLSs in whole protein sequences of the *H. pylori* proteome. Both monopartite and bipartite NLSs in the *H. pylori* proteome were determined (Fig. 3)*.* Proteins with NLS cutoff values of 3.0-5.0 were reported to mostly target the nucleus of the host cell with monopartite NLSs (Table 3). Proteins with NLS cutoff values of 0-3.0 were mostly found to target the nucleus of the host cell with bipartite NLSs (Table 3).

### Prediction of protein targeting using the BaCelLo predictor

A total of 85 (out of 1,552) proteins in the *H. pylori* proteome were predicted to target the nucleus of the host cell using the BaCeILo predictor 29. The details of *H. pylori* proteins that target the host cell nucleus based on various parameters are shown in Table 4.

### Selection of BaCeILo-predicted proteins using the Hum-mPLoc 2.0 predictor

Only 46 proteins were consistently shown to target the nucleus of the host cell by the software of Hum-mPlooc 2.0 30. Fig. 2 and Fig. 3 illustrate the patterns of *H. pylori* proteins targeting host cell nucleus according to different parameters. Moreover, the details of the 46 proteins along with their functions are shown in Table 4.

## Discussion

Various studies have revealed the different possible factors involved in the development of cancer, including genetic factors, gender, age, diet, consumption of tobacco, inflammation, and infections by various pathogens. Infection is considered a leading factor involved in the development of about 16% of cancers 33. It has confirmed that various specific bacterial strains have the ability to alter numerous pathways and molecular events in the host cell for their own survival and involved in the growth and development of different types of cancer 10, 32, 34. In a report Arthur et. al (2012) demonstrated the involvement of *E. coli* NC101 strain in the progression of invasive carcinoma in azoxymethane (AOM)-treated Il10(-/-) mice. We have illustrated the involvement of *mycoplasma hominis* and *Chlamydia pneumoniae* protein targeting and their implication in the progression of prostate cancer and lungs cancer in recently published study 31, 32, 34. It was only in the early 1990s that the role of H. pylori as a causative agent of cancer was highlighted 35. The molecular mechanisms underlying gallbladder carcinogenesis remain unclear even today. We have proposed that various nucleus-targeting proteins of H. pylori alter the normal function of host cells. pI values failed to explain the pattern for nuclear targeting (Table 2). The association between H. pylori proteins that targeted the host cell nucleus and various parameters is shown in Fig. 2. The process of targeting the host nucleus is a key event that involves the regulation of the host cell. This is generally analyzed through specific motifs in protein sequences called NLSs. The NLS predictor allows prediction of the possible activity of an NLS in the amino acid sequences of different proteins. Various predictors may analyze the specific motifs in the amino acid sequences. The NLS mapper identified six classes of NLSs such that the nuclear import proteins are transported through the α/β pathways of importin. Therefore, we utilized the NLS mapper in our study to predict NLS activity in both monopartite and bipartite NLSs (enriched basic amino acid stretches) 36. The NLS predictor identified potential localization sites of the proteins, including the nucleus, partially in the nucleus, the cytoplasm, and equally in both the cytoplasm and nucleus of the host cell.

In addition, BaCeILo and Hum-mPLoc 2.0 were employed in the current study to analyze H. pylori protein targeting to different host cell compartments. The results of BaCeILo and Hum-mPLoc 2.0 revealed the variation in protein targeting to the nucleus because of the utilization of different datasets during prediction. Such differences in the results from various predictors are acceptable. The Hum-mPLoc predictor analyzes the targeting of proteins to different compartments of cells using sequential evolution and domain information. The predictor computes 14 subcellular compartments such as the nucleus, mitochondrion, cytoplasm, endoplasmic reticulum, lysosome, Golgi apparatus, plasma membrane, and peroxisome. Proteins with MW < 40 kDa may be transported to the nucleus through passive transport mechanisms 28. In the present study, we predicted various H. pylori proteins with MW < 40 kDa that affected the normal pathways of cells and may be involved in the progression of GBC. Furthermore, the nucleus-targeting proteins in humans determined by Hum-mPLoc 2.0 were compared with those determined by BaCelLo to more accurately define the subcellular localization of *H. pylori* proteins. The Hum-mPLoc 2.0 predictor confirmed only 46 H. pylori proteins that were targeted to the nuclei of host cells.

We focused on evaluating the involvement of the nucleus-targeting proteins of H. pylori in the progression and development of GBC. H. pylori-derived effector proteins may alter the host cell internal environment through the induction of immunosuppression, suppression of tumor suppressor genes, activation of chronic inflammation, and transformation of normal cells 37.

### H. pylori proteins that target the nuclei of host cells and their implications in GBC

From the whole H. pylori proteome with 1,552 proteins, only 46 proteins were predicted to be targeted to the host cell nucleus during intracellular infection. This specific targeting may alter the homeostasis of normal cells. The results of the current study should be validated through experimental research in wet laboratories prior to drawing any final conclusions. The corresponding results may be used to develop therapies to manage and cure cancer.

### Replication, DNA binding, and DNA repair in the development of GBC

Various factors such as genomic instability determine cancer susceptibility. However, the molecular mechanisms that lead to the development of cancer are incompletely understood. A report showed that H. pylori infection suppressed the expression of p53 protein 38. A prominent hypothesis is that alterations in replication or the establishment of error-prone DNA synthesis phenotypes originating in genomic instability may serve as a source of cancer 39. Progression of cancer is affected by different DNA-binding proteins such as the methyl CpG-binding protein, which detects the methylation of DNA and its components. Together these proteins play an important role in the development of cancer 40. Furthermore, the tightly controlled DNA replication is essential for the multiplication of normal cells, and mutations in proteins involved in DNA replication have been associated with the development of different types of cancers 41, 42.

Diverse DNA-binding proteins have been predicted to target the nuclei of host cells, including DNA topoisomerase 1 (accession no. P55991), DNA polymerase III subunit beta (accession no. O25242), ribonuclease R (RNase R) (accession no. P56123), DNA topoisomerase (accession no. O25188), DNA polymerase III gamma and tau subunits (accession no. O25419), and the IS200 insertion sequence from SARA17 (accession no. O34550). These nucleus-targeting proteins and other uncharacterized proteins may affect the replication process in the nucleus of the host cell. The functions and other details of these proteins are shown in Table 4. Bacterial insertion sequences IS200 and IS607 encode a transposase (TnpA) and one protein with unknown function (TnpB) that is believed to act as a methyltransferase 43. The levels of methyltransferase are increased in some cancer cell lines and cancer tissues, wherein these enzymes may be involved in the hypermethylation of the promoter CpG-rich regions of the tumor suppressor genes 44.

### Transcription and translation regulatory proteins in the development of GBC

The progression from normal to cancerous cells is associated with alterations in protein-protein interactions, either in the transcription or translation regulatory proteins. The dysregulation in the expression of various genes may lead to the suppression of different anti-oncogenes and activation of proto-oncogenes during bacterial infection 45. Conserved structural similarities in different subunits of RNA polymerase as well as antigenicity are specific features of eukaryotes. The current study showed that H. pylori RNA polymerase sigma factor RpoD (accession no. P55993), response regulator (accession no. O25684), and transcription termination/antitermination protein NusG (accession no. P55976) target the host cell nucleus and may alter the normal pathways in the host cell. The unfolded response regulator has been reported as a new predictive biomarker for the identification of cancers 46. Nevertheless, the possible involvement of such proteins in the dysregulation of normal pathways must be experimentally demonstrated before making final conclusions.

Various H. pylori translation regulatory proteins similarly target the host cell nucleus, including translation initiation factor IF-3 (accession no. P55973), ribosome-recycling factor (RRF) (accession no. P56398), and 30S ribosomal protein S8 (accession no. P66621). These proteins also disturb the normal functioning of protein synthesis by altering gene expression. Alterations in gene expression may lead to the progression of GBC.

### Uncharacterized proteins in the development of GBC

Various uncharacterized H. pylori proteins were predicted to target the nucleus of the host cell, including Cag pathogenicity island protein (Cag7) (accession no. O25262), Cag pathogenicity island protein (Cag10) (accession no. O25265), and another uncharacterized protein (accession no. O25010). These proteins may also act as factors that promote carcinogenesis in the gallbladder. For instance, CagA may interact with a tumor suppressor protein (RUNX3) that is commonly inactivated in gastric carcinomas 47.

## Conclusions

The current work examines the mechanisms underlying the progression of GBC during *H. pylori* infection and the possible implications of the nucleus-targeting proteins in the development of GBC. The novel findings of this study may suggest new approaches to manage and cure GBC.

## Figures and Tables

**Table 1:** *In silico* analysis of *Helicobacter pylori* proteins targeted to the host cell nuclei their relation to all proteins with similar molecular weight

S. no.	Molecular weight (kW)	Number of proteins targeting nucleus	Total number of proteins	Percentage
1	0-20	14	434	3.22
2	20-40	15	602	2.49
3	40-60	9	326	2.76
4	60-80	5	105	4.76
5	> 80	3	86	3.48

**Table 2:** *In silico* analysis of *H. pylori* proteins targeted to the host cell nucleus and their relation to all proteins with similar pI value

S. no.	Isoelectric point	Number of proteins targeting nuclei	Total number of proteins	Percentage
1	3.0-5.0	5	64	7.81
2	5.0-6.0	6	273	2.19
3	6.0-7.0	5	245	2.04
4	7.0-8.0	6	127	4.72
5	8.0-9.0	14	330	4.24
6	9.0-10.0	10	450	2.22
7	10.0-11.0	0	52	0
8	11.0-13.0	0	12	0

**Table 3:** *In silico* analysis of *H. pylori* proteins targeted to the host cell nucleus and their relation to all proteins with similar monopartite and bipartite nuclear localization signals (NLS)

NLS	NLS cut-off	Number of proteins targeting nucleus	Total number of proteins	Percentage
Monopartite	0-3.0	5	1377	0.36
Monopartite	3.0-5.0	22	101	21.78
Monopartite	5.0-8.0	19	62	30.64
Monopartite	> 8.0	0	13	0
Bipartite	0-3.0	42	271	15.49
Bipartite	3.0-5.0	2	827	0.24
Bipartite	5.0-8.0	1	441	0.22
Bipartite	> 8.0	1	14	7.14

**Table 4:** Descriptions of Helicobacter pylori proteins targeted into the nucleus of host cells as predicted using various tools

Accession number	Protein name	Function in bacteria	Evidence of protein	Isoelectric point	Molecular weight in daltons	NLS Mapper Monopartite Bipartite		BaCeILo	Hum-mPLoc 2.0
P55973	Translation initiation factor IF-3	Translation initiation factor activity	Protein inferred from homology	9.57	23342	0	4.9	**Nucleus**	**Nucleus**
P56131	tRNA-2-methylthio-N(6)-dimethylallyladenosine synthase (EC 2.8.4.3) (Dimethylallyl)adenosine tRNA methylthiotransferase MiaB) (tRNA-i(6)A37 methylthiotransferase)	4 iron, 4 sulfur cluster binding, metal ion binding, transferase activity	Protein inferred from homology	8.75	49423	0	5.5	**Nucleus**	**Nucleus**
O25029	DEAD-box ATP-dependent RNA helicase RhpA (EC 3.6.4.13)	ATP binding, helicase activity, RNA binding	Experimental evidence at the protein level	8.78	55806	2	6.3	**Nucleus**	**Nucleus**
P56398	Ribosome-recycling factor (RRF) (Ribosome-releasing factor)	Translation	Protein inferred from homology	7.79	20915	0	3.7	**Nucleus**	**Nucleus**
O26061	Flavin-dependent thymidylate synthase (FDTS) (EC 2.1.1.148) (FAD-dependent thymidylate synthase) (Thymidylate synthase ThyX) (TS) (TSase)	Flavin adenine dinucleotide binding, thymidylate synthase (FAD) activity	Experimental evidence at the protein level	6.76	24071	0	3.2	**Nucleus**	**Nucleus**
P55991	DNA topoisomerase 1 (EC 5.99.1.2) (DNA topoisomerase I) (Omega-protein) (Relaxing enzyme) (Swivelase) (Untwisting enzyme)	DNA binding, DNA topoisomerase type I activity, metal ion binding	Experimental evidence at the protein level	9.04	83196	2	4.9	**Nucleus**	**Nucleus**
P55986	Uncharacterized RNA pseudouridine synthase HP_1459 (EC 5.4.99.-) (RNA pseudouridylate synthase) (RNA-uridine isomerase)	Pseudouridine synthase activity, RNA binding	Protein inferred from homology	9.9	30228	3	6.8	**Nucleus**	**Nucleus**
O25506	DNA-binding protein HU	DNA binding	Protein inferred from homology	9.05	10384	0	5.8	**Nucleus**	**Nucleus**
O25242	DNA polymerase III subunit beta (EC 2.7.7.7)	3'-5' exonuclease activity, DNA binding, DNA-directed DNA polymerase activity	Experimental evidence at the protein level	5.51	42185	0	4.2	**Nucleus**	**Nucleus**
P57798	Putative Fe (2+) transport protein A	Ion transport, iron, ion homeostasis	Protein inferred from homology	8.62	8719	0	5.8	**Nucleus**	**Nucleus**
O25929	Flagellar assembly factor FliW 2	Bacterial-type flagellum assembly, regulation of translation	Experimental evidence at the protein level	5.92	14862	0	2.8	**Nucleus**	**Nucleus**
P55976	Transcription termination/antitermination protein NusG	DNA-templated transcription, termination, regulation of DNA-templated transcription, elongation, transcription antitermination	Protein inferred from homology	6.98	20261	0	2.9	**Nucleus**	**Nucleus**
P55993	RNA polymerase sigma factor RpoD (Sigma-70)	DNA binding, DNA binding transcription factor activity, sigma factor activity	Experimental evidence at the protein level	7.94	77737	4	5.2	**Nucleus**	**Nucleus**
P56123	Ribonuclease R (RNase R) (EC 3.1.13.1) (VacB protein homolog)	Exoribonuclease II activity, RNA binding	Protein inferred from homology	9.17	74163	0	4.4	**Nucleus**	**Nucleus**
P66621	30S ribosomal protein S8	rRNA binding, structural constituent of ribosome	Protein inferred from homology	9.78	15184	0	4.3	**Nucleus**	**Nucleus**
O25475	Protein translocase subunit SecA	ATP binding, metal ion binding	Protein inferred from homology	5.62	99084	0	5.6	**Nucleus**	**Nucleus**
Q09064	Urease accessory protein UreE	Nickel cation binding	Experimental evidence at the protein level	8.58	19382	0	5.4	**Nucleus**	**Nucleus**
O25448	Flagellar protein FliS	Bacterial-type flagellum assembly	Experimental evidence at the protein level	5.03	14543	0	0	**Nucleus**	**Nucleus**
O25684	Response regulator	DNA binding	Experimental evidence at the protein level	5.22	25468	0	3.1	**Nucleus**	**Nucleus**
O25998	Secreted protein involved in flagellar motility	Unknown	Experimental evidence at the protein level	6.54	20480	3	3.3	**Nucleus**	**Nucleus**
O25262	Cag pathogenicity island protein (Cag7)	Unknown	Protein predicted	5.61	219401	2.5	6.8	**Nucleus**	**Nucleus**
O25085	Uncharacterized protein	Unknown	Protein predicted	6.74	15304	0	5.4	**Nucleus**	**Nucleus**
O24903	Uncharacterized protein	Unknown	Protein predicted	5.24	57408	8	7.5	**Nucleus**	**Nucleus**
O25555	Uncharacterized protein	Unknown	Protein predicted	8.8	14714	0	5	**Nucleus**	**Nucleus**
O25800	Uncharacterized protein	Unknown	Protein predicted	9.45	30632	0	4.4	**Nucleus**	**Nucleus**
O25576	Uncharacterized protein	Metal ion binding	Protein predicted	10.13	16137	0	3.3	**Nucleus**	**Nucleus**
O25834	Uncharacterized protein	Unknown	Protein predicted	5.57	22149	5	5.7	**Nucleus**	**Nucleus**
O24939	Uncharacterized protein	Unknown	Protein predicted	9.54	45264	2	4.3	**Nucleus**	**Nucleus**
O24937	Uncharacterized protein	Unknown	Protein predicted	9.46	45136	0	3.9	**Nucleus**	**Nucleus**
O25553	Uncharacterized protein	Unknown	Protein predicted	9.79	11356	0	6.6	**Nucleus**	**Nucleus**
O25652	Conjugal transfer protein (TraG)	Unidirectional conjugation	Protein predicted	8.89	20442	0	4	**Nucleus**	**Nucleus**
O25650	Uncharacterized protein	Unknown	Protein predicted	8.76	32600	0	6.5	**Nucleus**	**Nucleus**
O25105	Uncharacterized protein	Unknown	Protein predicted	9.81	21978	0	4.5	**Nucleus**	**Nucleus**
O25188	DNA topoisomerase (EC 5.99.1.2)	DNA binding, DNA topoisomerase type I activity	Protein inferred from homology	8.47	77677	0	5.8	**Nucleus**	**Nucleus**
O25799	Uncharacterized protein	Unknown	Protein predicted	9.56	43994	0	3.5	**Nucleus**	**Nucleus**
O25195	Uncharacterized protein	Unknown	Protein predicted	5.36	41437	0	5.1	**Nucleus**	**Nucleus**
O24905	Uncharacterized protein	Unknown	Protein predicted	4.73	14086	0	2.7	**Nucleus**	**Nucleus**
O25102	Uncharacterized protein	Unknown	Protein predicted	6.56	6989	0	2.1	**Nucleus**	**Nucleus**
O25304	Uncharacterized protein	Transport	Protein predicted	9.46	37382	0	4.3	**Nucleus**	**Nucleus**
O25419	DNA polymerase III gamma and tau subunits (DnaX)	ATP binding, DNA-directed DNA polymerase activity	Protein predicted	5.87	66245	0	4.8	**Nucleus**	**Nucleus**
O25379	Uncharacterized protein	ATP binding, DNA binding, hydrolase activity	Protein predicted	8.05	68760	10.5	6.4	**Nucleus**	**Nucleus**
O25131	Uncharacterized protein	Unknown	Protein predicted	7.85	23211	0	6.1	**Nucleus**	**Nucleus**
O25010	Uncharacterized protein	Regulation of transcription, DNA-templated	Protein predicted	9.52	8599	0	4.2	**Nucleus**	**Nucleus**
O25373	Uncharacterized protein	Unknown	Protein predicted	8.93	47633	0	4.7	**Nucleus**	**Nucleus**
O25265	Cag pathogenicity island protein (Cag10)	Unknown	Protein predicted	9.54	29095	0	5.9	**Nucleus**	**Nucleus**
O34550	IS200 insertion sequence from SARA17	DNA binding, transposase activity	Protein predicted	8.44	15965	0	4.6	**Nucleus**	**Nucleus**

## Abbreviations

*H. pylori*: *Helicobacter pylori*
GBC: Gallbladder cancer
NLS: Nuclear localization signal
BaCelLo: Balanced Subcellular Localization
Hum-mPLoc 2.0.: Human protein subcellular localization
UniProt: Universal Protein Resource
pI: Isoelectric point
MW: Molecular weight
